# A case of severe leptospirosis with Jarisch–Herxheimer reaction presenting as respiratory failure

**DOI:** 10.3389/fpubh.2023.1125306

**Published:** 2023-02-10

**Authors:** Yunzhen Shi, Wanru Guo, Ming Hu, Yuxuan Wang, Jingnan Li, Wenjuan Hu, Xiaomeng Li, Kaijin Xu

**Affiliations:** ^1^Department of Infectious Diseases, Affiliated Dongyang Hospital of Wenzhou Medical University, Dongyang, Zhejiang, China; ^2^State Key Laboratory for Diagnosis and Treatment of Infectious Diseases, National Clinical Research Center for Infectious Diseases, Collaborative Innovation Center for Diagnosis and Treatment of Infectious Diseases, The First Affiliated Hospital, Zhejiang University School of Medicine, Hangzhou, Zhejiang, China

**Keywords:** leptospirosis, Jarisch-Herxheimer reaction, pulmonary alveolar hemorrhage, metagenomic next-generation sequencing, imaging features

## Abstract

**Background:**

Leptospirosis is a widespread zoonotic disease caused by pathogenic *Leptospira* spp. The treatment of penicillin or tetracycline can cause a Jarisch–Herxheimer reaction (JHR), which can lead to acute respiratory distress syndrome (ARDS) and multi-organ failure in severe cases. The overall course of evolution and imaging features of a JHR exacerbation of leptospirosis have rarely been reported.

**Case presentation:**

We present a case of leptospirosis complicated by pulmonary alveolar hemorrhage and a Jarisch-Herxheimer reaction (JHR) that required respiratory and vasopressor support. This case demonstrates a well-defined course of evolution of JHR and the imaging features.

**Conclusions:**

Leptospirosis is easily misdiagnosed in some sporadic areas, and JHR complicates its management. Early diagnosis and appropriate treatment can reduce the mortality of severe leptospirosis with JHR.

## Introduction

Leptospirosis is a widespread zoonotic disease caused by pathogenic *Leptospira* spp. It is transmitted to humans through contact with the urine of infected mammals (especially mice) and is a significant threat to the global public health (1). It is endemic to tropical and subtropical regions, and was one of the critical infectious diseases to be prevented and controlled in southern China. In the last 30 years, the incidence of leptospirosis has decreased. In 2021, 403 cases were reported in China, making it a rare clinical infectious disease. It is easy to miss a diagnosis if the clinician does not understand the condition well. During treatment, penicillin or tetracycline can cause a Jarisch–Herxheimer reaction (JHR), which can lead to acute respiratory distress syndrome (ARDS) and multi-organ failure in severe cases, complicating the management of *leptospira* spp. (2). However, the overall course of evolution and imaging features of a JHR exacerbation of leptospirosis have rarely been reported. Here, we report a life-threatening case of leptospirosis with pulmonary alveolar hemorrhage (PAH) and JHR.

## Case presentation

**Figure 1:**
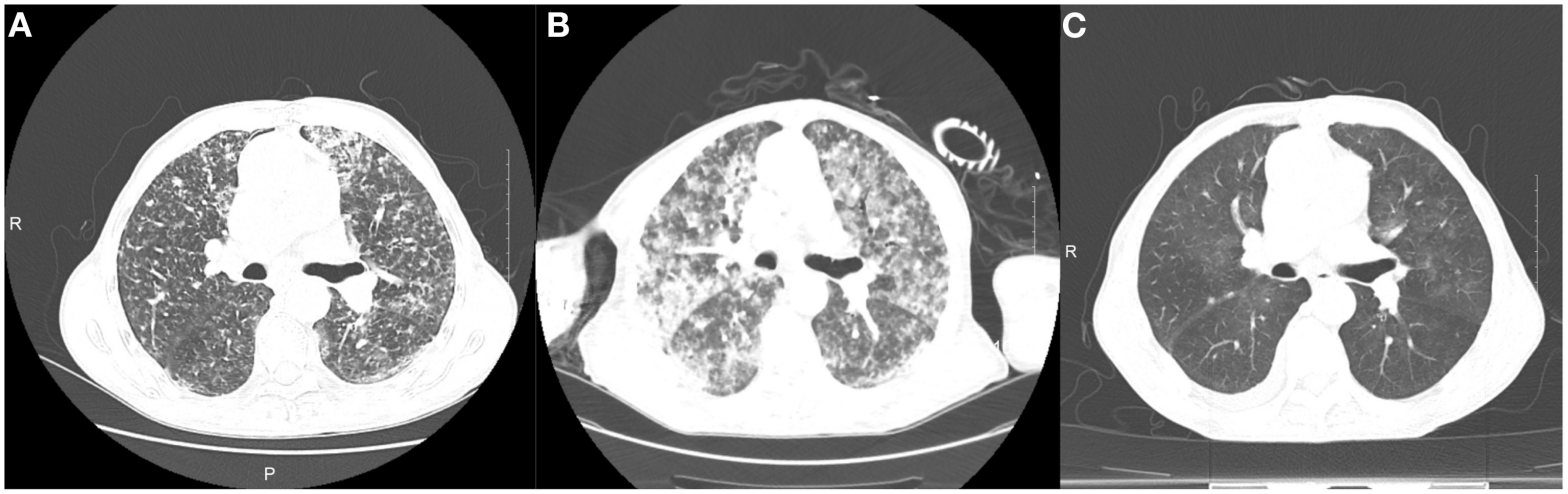
Computed tomography findings. Computed tomography images were respectively taken on admission **(A)**, after intravenous piperacillin tazobactam **(B)**, and before discharge **(C)**.

A 73-year-old male farmer presented to the emergency department with a 4-day history of fever (38.3°C), fatigue, chest tightness, and myalgia. On presentation, the patient was alert, with regular heart rate and normal blood pressure. Physical examination revealed a temperature of 39°C and gastrocnemius muscle pain impairing walking. Laboratory tests revealed thrombocytopenia with a platelet count of 48 × 10^9^/L. Renal function tests revealed an elevated creatinine level (237 umol/L). CPK was 791 U/L (normal range 50–310 U/L) and CK-MB was 26 U/L (normal range <25 U/L). The C-reactive protein level was 160.44 mg/dL, and his hemoglobin level, white blood cell count and live function test was normal. Initial computed tomography (CT) of the chest revealed multiple infections in both lungs (Figure 1A).

The patient was diagnosed with community-acquired pneumonia and started on intravenous piperacillin–tazobactam at 13:55. At 15:22, the patient developed unconsciousness, fever, rigor, increasing tachycardia with a heart rate of 190 beats/min, hypotension (82/42 mmHg), tachypnea with a respiratory rate of 36 breaths/min, and hypoxia with peripheral capillary oxygen saturation of 78%. Intubation for respiratory support was immediately performed, and bloody sputum was aspirated during suctioning. Despite fluid therapy, the patient's blood pressure remained low, and norepinephrine was administered to maintain blood pressure. A repeat chest CT showed diffuse bilateral exudation that had worsened after previous imaging (Figure 1B). His C-reactive protein level was elevated to 200.22 mg/L. Arterial blood gas analysis showed a PaO2 of 49.4 mm Hg and an oxygenation index of 80.98 with an oxygen concentration of 61%. Fiberoptic bronchoscopy revealed dark red, bloody sputum in the airways, and metagenomic next-generation sequencing (mNGS) samples collected from the airways and bronchoalveolar lavage fluid revealed *Leptospira interrogans*. Further history revealed that the patient had been exposed to and consumed raw river water before the onset of symptoms. The diagnosis of leptospirosis was made based on the patient's history, fiberoptic bronchoscopy results, CT manifestations, laboratory tests, and clinical presentation.

Additionally, the patient's disease state worsened dramatically after the administration of piperacillin tazobactam, with respiratory failure and septic shock. Pulmonary CT showed diffuse exudative lesions (examined 2.5 h after treatment), deemed JHR caused by penicillin treatment. The penicillin dose was reduced, and 40 mg methylprednisolone was administered every 12 h. When the conditions improved, the penicillin dose was adjusted to 400,000 units every 8 h for 2 weeks and subsequently discontinued. On the 7th day after onset, pulmonary CT showed an absorption of the lesion. Methylprednisolone was reduced after 2 weeks to 30 mg every 12 h for 3 days, 20 mg every 12 h for 3 days, and 20 mg daily for 3 days until discontinued.

After 2 weeks of treatment, the patient's temperature gradually decreased to normal, myalgia resolved, blood gas analysis showed a gradual increase in oxygenation index from 80.98 to 342.42 and 369.7 to normal, and chest imaging improved significantly (Figure 1C); norepinephrine was discontinued, and the endotracheal tube was removed.

## Discussion

Leptospirosis is a neglected and re-emerging disease of global public health importance. It is estimated that more than one million cases of leptospirosis occur worldwide each year, causing ~60,000 deaths (3). Severe icteric leptospirosis can cause jaundice, renal impairment and severe bleeding complications with a high mortality rate. Patient with leptospirosis pulmonary involvement may present with chest pain, cough, dyspnea, and hemoptysis, and in severe cases ARDS may develop (4). Studies have shown that dyspnea or pulmonary infiltration on chest radiography is a poor prognostic indicator of severe leptospirosis and is associated with mortality (5). ARDS has been described in patients with leptospirosis and its temporal association with JHR has been clarified (6). However, to our knowledge the overall course of JHR and its imaging characteristics have rarely been reported.

Early diagnosis is critical because leptospirosis is sporadic in parts of China and can be easily missed by clinicians. In our case, leptospirosis was not considered for the first time due to the inadequate vigilance of emergency physicians, and the diagnosis was not made early enough. Leptospirosis should be considered an emerging infectious disease in patients with fever, myalgia and lung involvement. Early detection of leptospirosis primarily depends on the patient's symptoms, signs, risk factors and contact history. In addition to blood, urine, and other body fluid cultures, conventional microscopic agglutination tests and enzyme immunoassays, mNGS is a novel method that helps in the early diagnosis of patients with atypical leptospirosis symptoms. It can also be used for typing, virulence and antibiotic resistance analyses. As in our case, *Leptospira* spp. was detected in bronchoalveolar lavage fluid using mNGS, which was helpful for clinical diagnosis and treatment.

Penicillin is the drug of choice for treating leptospirosis; however, cephalosporins, doxycycline and chloramphenicol are also options. JHR is a known complication that occurs during the treatment of spirochaete infections, including syphilis, leptospirosis, Lyme disease, louse-borne relapsing fever, and tick-borne relapsing fever. Syphilis carries the highest risk for the development of JHR. The occurrence of JHR in other spirochaete infections is as follows: Lyme disease (7–30%), leptospirosis (9%), tick-borne relapsing fever (39%), louse-borne relapsing fever (37–100%) (2). The antibiotics most related to the development of JHR are penicillin, tetracycline and erythromycin. Newer antimicrobials such as cephalosporins, meropenem, levofloxacin, ciprofloxacin, clarithromycin, and azithromycin can also cause JHR. It is characterized by the onset of fever, chills, rigors, myalgias, hypoxia, tachycardia, and hypertension or hypotension within 24 h of antibiotic therapy (7). JHR is thought to be caused by the release of endotoxin from spirochaete destruction and the activation of the cytokine cascade. The common complications of JHR include PAH, meningitis, cardiac injury, renal and liver dysfunction, worsening brain function and premature uterine contractions in pregnant women. It is important to differentiate JHR from antibiotic anaphylaxis because of the similar presentation. Previous drug reactions should be carefully evaluated, especially to penicillin. Generalized urticaria/rubella, eosinophilia, or the presence of penicillin-specific antibodies support the diagnosis of anaphylaxis (7). The patient in our case had no previous history of penicillin allergy, no adverse reactions in penicillin skin test before treatment, and had pulmonary hemorrhage, which is a common complication of JHR in leptospirosis but has not been reported after penicillin allergy. In our case, the CT findings showed a sudden deterioration after the initiation of antibiotic treatment, and the CT findings, with the patient's physical findings, were consistent with PAH. Therefore, we believe that PAH was caused by leptospirosis and exacerbated by JHR. In addition to leptospirosis, infectious diseases that cause PAH include influenza A, dengue, malaria, and Staphylococcus aureus infection, which should be carefully distinguished by clinicians (8). Two main mechanisms of PAH are suggested: a toxin-mediated mechanism and/or the immune responses of the host. The release of numerous toxic bacterial species leads to damage and defects in the basement membrane of pulmonary capillaries during antibiotic-mediated bacterial lysis; the linear deposition of immunoglobulins (IgA, IgG, and IgM) and complements on alveolar surface is also associated with the pathogenesis of PAH in leptospirosis (8, 9). A retrospective study found that delays <3 days between the onset of symptoms and the initiation of antibiotic therapy were an independent risk factors for the occur of JHR (10). This underscores the need to monitor patients when antimicrobial therapy is initiated early in their disease course. The treatment of JHR is mainly to control the inflammatory response and provide life support. Steroids and other immunomodulators can be used as adjuvant therapies when the inflammatory response to infection persists. Pulsed high-dose glucocorticoid therapy has been suggested to be beneficial in patients with severe pulmonary leptospirosis (11). Previous studies have shown that antibodies against tumor necrosis factor-α can decrease the prevalence of JHR (12).

In conclusion, we report a severe case of leptospirosis with PAH, which was exacerbated by JHR, and show the complete clinical evolution, diagnosis and treatment, as well as mNGS and CT findings. This case is an instructive reminder for clinicians to take a detailed medical history, consider the likelihood of leptospirosis and anticipate JHR before initiating antibiotics, so that supportive care can be optimized as soon as possible.

## Data availability statement

The original contributions presented in the study are included in the article/supplementary material, further inquiries can be directed to the corresponding author.

## Ethics statement

Written informed consent was obtained from the individual(s) for the publication of any potentially identifiable images or data included in this article.

## Author contributions

YS, WG, MH, and KX were involved in the conception and design of the study. YS, WG, MH, YW, JL, WH, and XL were involved in the collection and assembly of data. YS and WG wrote the manuscript. All authors contributed to the article and approved the submitted version.
